# Use of Implementation Mapping to Develop a *Promotora* Empowerment Training for a Faith-Based Physical Activity Program for Latinas

**DOI:** 10.1007/s11121-026-01949-5

**Published:** 2026-07-16

**Authors:** Jackelyne Garcia-Villegas, Melanie Gomez, Jennifer Schneider, Taynara Formagini, Oliva Lafuente, Borsika A. Rabin, Samantha M. Harden, Guadalupe X. Ayala, Ross C. Brownson, Elva M Arredondo

**Affiliations:** 1 SDSU/UC San Diego Joint Doctoral Program in Clinical Psychology, 5500 Campanile Dr, San Diego, CA 92182, USA; 2 Department of Psychology , San Diego State University , 5500 Campanile Dr, San Diego, CA 92182, USA; 3 Institute for Behavioral and Community Health , San Diego State University Research Foundation, 9245 Sky Park Court, Suite 221, San Diego, CA 92123, USA; 4 Department of Family Medicine, University of California, 9500 Gilman Dr, La Jolla, San Diego, CA 92093, USA; 5 The Herbert Wertheim School of Public Health and Human Longevity Science, University of California, 9500 Gilman Dr, La Jolla, San Diego, CA 92093, USA; 6 Department of Human Nutrition, Foods, and Exercise Virginia Tech, 1981 Kraft Drive, Blacksburg, VA 24060-000, USA; 7 School of Public Health , San Diego State University, 5500 Campanile Drive, San Diego, CA 92182, USA; 8 Prevention Research Center, Bursky School of Public Health at Washington University in St. Louis, 4300 Duncan Street, Suite 2121, St. Louis, MO 63130, USA; 9 Department of Surgery, Division of Public Health Sciences, and Alvin J. Siteman Cancer Center , Washington University School of Medicine, Washington University in St. Louis, St. Louis, MO, 63130, USA; 10 Department of Psychology, San Diego State University, 5500 Campanile Dr, San Diego, CA 92182, USA

**Keywords:** Physical activity intervention, Faith-based organization, Implementation Mapping, *Promotoras*

## Abstract

Physical activity (PA) interventions in faith-based organizations (FBOs) delivered by para-professionals (e.g., community health workers, *promotoras*) have been effective in increasing PA among participants. However, given their role within the FBO, *promotoras* often face implementation challenges such as limited organizational support and resources and lack of familiarity with how to change organizational procedures. Many existing *promotora* trainings lack professional skills development needed to address these barriers. This study developed a *promotora* empowerment training program to improve PA intervention implementation in FBOs and increase physical activity levels among Latina parishioners. The study used Implementation Mapping to develop a *promotora* empowerment training curriculum within the Faith in Action project, an evidence-based intervention designed to increase PA levels among Latina parishioners. Curriculum development included (1) conducting a needs assessment through post-intervention focus groups with Faith in Action *promotoras*; (2) identifying implementation outcomes and performance objectives, identifying determinants, and creating matrices of change objectives; (3) selecting theory-informed methods and implementation strategies; (4) developing implementation protocols and materials; and (5) planning for implementation evaluation. (1) Focus group findings suggest that *promotoras* needed additional training in advocating for resources and problem-solving barriers with FBO staff. (2) Key implementation outcomes (e.g., engaging FBO leaders), performance objectives (e.g., identifying resources), and the determinants (e.g., understanding program goals) influencing these objectives were identified. Change objectives were mapped to the corresponding performance objectives and determinants. (3) Empowerment Theory and Dialectical Behavior Therapy interpersonal skills were selected to guide the development of the training. (4) The study team focused training sessions on communication, conflict resolution, planning, problem-solving, and social media use. The training will be delivered by a *promotora* coordinator in Spanish. (5) Implementation outcomes will be evaluated by examining program fidelity and participant engagement. This study offers a roadmap for development of future *promotora* trainings and can inform the extent to which the additional *promotora* training leads to successful implementation outcomes and enhanced efficacy. This study also serves as an example of how Implementation Mapping can be utilized to improve implementation outcomes for a high-priority population in real-world settings.

Adult Latinas experience a disproportionate burden of cardiovascular disease (Lloyd-Jones et al., 2010), obesity (Center for Disease Control & Prevention, 2024), and diabetes (Cordero et al., 2022). Engaging in regular physical activity (PA) is essential for both managing and preventing these health conditions (Centers for Disease Control & Prevention, 2024). However, Latinas engage in very little PA, with only a third adhering to the recommended US national guidelines for moderate to vigorous PA (Arredondo et al., 2016) compared with 59% among all adult women (Du et al., 2019). Multiple barriers, such as socioeconomic factors, cultural factors, and lack of access to exercise promoting environments, contribute to these health disparities (Tovar et al., 2018). Beyond structural barriers to physical activity, experiences of discrimination and acculturative stress have been examined as sociocultural determinants of physical activity engagement among Latinas, although findings across studies are mixed (Benitez et al., 2016; Luna et al., 2024). Discrimination and acculturative stress are consistently linked to psychological distress (Andrade et al., 2021), which may indirectly influence engagement in community-based physical activity programs (Schuch et al., 2017). Interventions delivered by trusted community members and implemented in culturally familiar settings may help mitigate sociocultural barriers to participation and support sustained engagement in physical activity through increased social support (Salinas et al., 2018). Culturally based interventions that utilize local community centers or similar accessible facilities have been shown to significantly increase PA participation among their members (Arredondo et al., 2022; Merzel & D’Afflitti, 2003). These settings offer numerous advantages because they are familiar and accessible, thus reducing barriers to participation, and potentially increasing interventions’ reach and efficacy, including representation and representativeness (Fort et al., 2023).

In our study, faith-based organizations (FBOs) present an optimal environment for PA interventions due to their strong community ties, trust among members, and established social support networks (Tristão Parra et al., 2018). FBOs provide a supportive setting that may enhance the implementation of health programs due to several factors, among them: the FBO’s mission to foster physical, emotional, and spiritual well-being (Haughton et al., 2020), its longstanding and recognizable presence within communities (Haughton et al., 2020), the physical space for supporting PA initiatives (Bernhart et al., 2020), and the communal support from fellow parishioners and clergy (Haughton et al., 2020; Majee et al., 2023). Given that the majority of US Latinos identify as either Catholic (43%) or Protestant (15%), with 22% of Catholics and 37% of Protestants attending weekly services (Pew Research Center, 2023), faith-based health initiatives hold potential for high reach and engagement of Latinas. However, despite their effectiveness (DeHaven et al., 2004), FBOs face barriers to effective program implementation, including the FBO’s culture of wellness, challenges in participant recruitment, the level of pastor involvement, and the ability to secure placement on the FBO calendar (Tettey, 2018). Further research is needed to explore the best strategies for implementing health promotion programs in FBOs (Haughton et al., 2020). Identifying strategies to implement these programs can increase program reach, relevance, and sustainability (Proctor et al., 2023). However, the compilation of implementation strategies has often been established and tested in clinical settings; therefore, a community-based and community-informed approach to what implementation strategies are needed represents an emergent target of dissemination and implementation science (Balis et al., 2024).

One possible solution is through the involvement of *promotoras. Promotoras*, also referred to as community health workers, are trusted individuals who share cultural, linguistic, and lived experiences with the communities they serve (Ingram et al., 2012). They are often trained to promote health and prevent disease through the provision of social support. *Promotora* models differ depending on the organizations involved and funding available to support their efforts (Early et al., 2016; Gustafson et al., 2025). In some cases, they are already part of an organization delivering the intervention (Ayala et al., 2015), they may become embedded within organizations as a result of their involvement (Arredondo et al., 2015), or they may serve as external partners supporting implementation efforts (Borelli et al., 2021). *Promotoras* have also played an important role as implementers of community health initiatives, including those implemented in FBOs (Arredondo et al., 2022; Salinas & Parra-Medina, 2019), and have successfully collaborated with organizations to deliver health interventions to marginalized communities globally, including group-based programs, home visits, and clinic-based support (May & Contreras, 2007; Stacciarini et al., 2012). Their deep connections to the community uniquely position them to effectively deliver health promotion initiatives and address the specific needs of the populations they serve.

Although *promotoras* are typically members of the communities they serve and share cultural backgrounds with many participants (Ingram et al., 2012), they often work within organizational contexts that serve culturally diverse populations and operate under distinct institutional cultures. Some faith-based organizations, for example, serve congregations that include diverse racial and ethnic groups, each with differing cultural norms, health beliefs, and expectations regarding programming and engagement (Diocese of San Diego, 2026; Smith et al., 2025). In these contexts, *promotoras* may need to balance culturally grounded approaches tailored to Latina participants with broader organizational priorities and norms that reflect the diversity of the congregation. In addition, *promotoras* may differ culturally from organizational leaders and decision-makers, including clergy or administrative staff, particularly in language use, communication styles, power dynamics, and expectations related to health behaviors (Bopp & Fallon, 2011; Haughton et al., 2020; Orpinas et al., 2021). These differences may influence *promotoras*’ ability to advocate for program adaptations, negotiate roles, or communicate community needs within organizational hierarchies (Haughton et al., 2018). Beyond interpersonal cultural differences, *promotoras* must also navigate institutional culture, including formal policies, decision-making processes, scheduling constraints, and accountability structures that shape how programs are approved, adapted, and delivered (Haughton et al., 2020). Together, these multilayered cultural dynamics may influence implementation processes and require *promotoras* to engage in cross-cultural communication, role negotiation, and organizational navigation to support effective and sustainable program delivery. Additionally, when implementing change strategies that involve organizational changes, *promotoras* may encounter challenges in navigating implementation barriers due to competing priorities within the organization, limited resources, poor communication within organization, insufficient understanding of organizational procedures, and staff turnover (Early et al., 2016; Otiniano et al., 2012; Sherman et al., 2017). In fact, research has shown that these challenges significantly influence whether *promotoras* can successfully implement and sustain interventions within an organization (Koskan et al., 2013b) and have been identified as barriers to the sustainment of *promotora*-led programs (Koskan et al., 2013b, c).

Most *promotora* trainings focus on training *promotoras* in their connection and role serving community members, such as cultural competence, patient assessment, patient navigation, health education and promotion, and disease prevention (Covert et al., 2019; Koskan et al., 2013a; Plescia et al., 2008). These programs often lack comprehensive training in the professional skills necessary for implementing health programs within complex organizations, such as organizational skills, interpersonal communication skills, advocacy, leadership, and navigating institutional structures (Covert et al., 2019; Lee et al., 2023). Professional skill development for *promotoras* may not only benefit the specific program being delivered but may better equip *promotoras* to implement and sustain health interventions in their communities, ultimately leading to more successful health outcomes.

To address the existing gaps in *promotora* training content, a *promotora* empowerment training was developed using Implementation Mapping (Fernandez et al., 2019). Implementation Mapping is a five-step, systematic, theory-informed process that integrates implementation science with health promotion principles to guide the development of implementation strategies. This process involves specifying implementation actors, identifying performance objectives, and linking contextual barriers and facilitators to targeted, evidence-based strategies to produce implementation protocols and materials. Using Implementation Mapping, the *promotora* empowerment curriculum was designed to enhance the implementation of evidence-based PA interventions within FBOs (Fernandez et al., 2019). This systematic approach integrates implementation science with health promotion principles to develop a curriculum that will equip *promotoras* with the necessary skills and knowledge to navigate organizational and interpersonal challenges, tailor the program to meet the community’s needs, and to create meaningful change. By strengthening *promotoras*’ capacity to navigate organizational challenges and adapt interventions to community needs, this training aims to improve the efficacy and sustainability of physical activity programs for Latinas, ultimately supporting better health outcomes.

## Methods

### Study Design

This study describes the development of a *promotora* empowerment training curriculum for the second iteration of Faith in Action (i.e., Faith in Action II), an evidence-based PA intervention delivered through FBOs in San Diego County (Arredondo et al., 2022). Faith in Action is a multilevel intervention aimed at increasing PA by targeting individual-level factors (e.g., PA beliefs and motivation through support calls), interpersonal-level factors (e.g., enhancing social support through group PA classes), and organizational-level factors (e.g., bolstering program support through training of FBO leaders). Eligible churches were located in the San Diego area and offer at least one weekly Spanish-language service, ensuring reach to Latina congregants. With the support of the research team, participating churches recruit *promotoras* from within their congregations, who serve as the primary implementers of the intervention. As part of the intervention, *promotoras* lead six free group-based physical activity classes per week, including aerobic dance, strength training, and walking groups, offered at varying times. In addition to group classes, *promotoras* provide *llamadas de apoyo* (support calls) to enrolled participants using motivational interviewing techniques to identify and problem-solve barriers to PA participation. Faith-in-Action has been shown to increase PA levels among Latinas attending FBOs in San Diego, CA (Arredondo et al., 2022). Faith in Action II is a follow-up implementation-effectiveness trial aimed at enhancing and sustaining the implementation of the intervention in FBOs (Arredondo et al., 2026). Prior published outcomes demonstrated the effectiveness of Faith in Action in increasing physical activity among Latina participants (Arredondo et al., 2022). In addition, post-intervention focus groups and interviews with FBO leaders and *promotoras* identified key organizational and implementation barriers, highlighting the need for supplementary implementation strategies to support program delivery and sustainment (Haughton et al., 2018, 2020).

Faith in Action II hired and trained lay women from each FBO as paid *promotoras* to offer free group PA classes at their corresponding church. Using FBO pulpit announcements, personal recommendations, and word of mouth, research staff collaborated with FBO leaders to identify potential *promotoras*—Spanish-speaking women who were involved in the FBO and passionate about PA and/or health promotion. Eligible candidates were interviewed in-person to assess their leadership potential, their commitment to the two-year project, and their knowledge of the FBO and the local community. Once selected, *promotoras* underwent a six-week training that included CPR training, Zumba certification, and 20 hours of small group training sessions during which the *promotora* coordinator equipped *promotoras* with the skills to lead PA classes, conduct motivational phone calls, and distribute educational materials. The *promotora* empowerment training described in this paper was developed as an addition to the standard *promotora* training for Faith in Action II as part of a follow-up trial aimed at enhancing and sustaining the implementation of the Evidence Based Intervention. The San Diego State University’s Institutional Review Board approved all study procedures, including participant recruitment.

### Planning and Procedures

Implementation Mapping was used to plan and develop the *promotora* empowerment training. Implementation Mapping is a systematic process rooted in the principles of Intervention Mapping (Fernandez et al., 2019). It comprises of five essential steps that combine implementation science and health promotion to understand factors influencing implementation and guide the development of strategies to promote the adoption, use, and sustainability of interventions (see Fig. 1). To guide the Implementation Mapping process for the *promotora* empowerment training, a group of core study personnel involved in the design, coordination, and implementation of the Faith in Action II study was assembled. The core study personnel consisted of a doctoral student (JG), a master’s-level student (MG), the study Principal Investigator (EA), and the project manager (JS). The doctoral student led the day-to-day execution of Implementation Mapping tasks with guidance and oversight from the Principal Investigator and project manager, while the master’s-level student contributed to data synthesis and material development. The core study personnel met regularly throughout the training development process, with weekly meetings to collaboratively review findings, make decisions related to each task, and refine training components. The core study personnel’s ongoing collaboration ensured alignment between empirical findings, theoretical frameworks, and the practical realities of implementation within faithbased organizations.

#### Task One: Conduct an Implementation Needs Assessment

The team conducted an implementation needs assessment to identify barriers, facilitators, and strategies for successful implementation of Faith in Action. Post-intervention focus groups were conducted with Faith in Action *promotoras* (*n* = 10) who facilitated the program from 2010 to 2016. The Principal Investigator (EA) and one other research staff (JS) conducted three in-person focus groups in Spanish two years following the completion of the study using a semi-structured focus group guide developed using the Consolidated Framework for Implementation Research (CFIR; Damschroder et al., 2009). CFIR was chosen because it is comprehensive and well suited for examining domains influencing implementation effectiveness in complex, multilevel interventions. CFIR constructs are organized into five domains: intervention characteristics, outer setting, inner setting, characteristics of individuals, and process. The research team selected constructs within each domain that were most relevant to the implementation of group-based interventions in community settings and adapted the language to make it appropriate for laypeople in the FBO context (e.g., integrate Catholic vocabulary such as parishioner and priest). Each focus group lasted two hours and included 3–4 *promotoras* from different FBOs. Focus groups were audio-recorded, transcribed verbatim, reviewed by the interviewer to ensure accuracy, and translated from Spanish to English using standard protocols. Three initial coders, including a qualitative methods expert who provided training to the team, analyzed the first transcript using an iterative-deductive approach; each inter-pedently coded the transcript before discussing as a group to reach consensus and develop preliminary codebooks based on CFIR constructs (Haughton et al., 2018). Four total coders then used the initial codebook to independently code subsequent focus groups. All transcripts were coded by hand and input into Dedoose software for organization (Dedoose Version 9.0.17, 2021). The team revised the codebook as needed to add emerging codes, refine definitions, and sort into categories and themes. Coders would re-code the transcripts if necessary, after clarifying nuances in code definitions.

#### Task Two: Identify Adoption and Implementation Outcomes, Performance Objectives, Determinants, and Change Objectives

The team built upon the insights gathered from focus groups conducted in task 1 to identify adoption and implementation outcomes, determinants, and performance objectives, and develop matrices of change objectives. This process was conducted collaboratively by the core study personnel through a series of discussions and regular team meetings. Focus group data were reviewed by the planning team to identify recurring barriers and facilitators related to *promotoras*’ ability to deliver and sustain the intervention within faith-based organizations. These barriers and facilitators informed the specification of implementation outcomes, defined as the desired implementation-related behaviors of key actors. These implementation outcomes were mapped into corresponding performance objectives, defined as the specific, observable actions required of *promotoras* to achieve successful implementation.

Performance objectives were then mapped to determinants, defined as modifiable factors influencing whether *promotoras* are able to carry out the specified implementation actions. Barriers and facilitators were also mapped to commonly used determinant categories in Implementation Mapping, including knowledge, skills, self-efficacy, outcome expectations, and perceived organizational support. Determinants were selected based on their prominence across focus groups, their relevance to factors that could be addressed through training, and their alignment with the *promotora* role and the organizational context of faith-based organizations. For each performance objective, the planning team developed matrices of change objectives by crossing performance objectives with selected determinants. Change objectives specified the discrete changes required within each determinant to enable *promotoras* to successfully perform the performance objective.

#### Task Three: Select Theoretical Methods and Design Implementation Strategies

The research team selected theory-informed methods to address the key determinants identified in task 2. Selection of change methods and practical applications was conducted collaboratively by the planning team through iterative discussion and consensus. Guided by the Implementation Mapping Taxonomy of Change Methods (Kok et al., 2016), the team identified change methods with demonstrated relevance for influencing these determinants, such as modeling and guided practice. The Implementation Mapping Taxonomy of Change Methods outlines theory- and evidence-based techniques for influencing modifiable determinants of implementation behavior and provides guidance for translating these methods into contextually appropriate practical applications (Fernandez et al., 2019; Kok et al., 2016). These methods were then translated into practical applications and implementation strategies tailored to the *promotora* role and the faith-based organizational setting.

#### Task Four: Develop Implementation Protocols and Materials

Task 4 of the Implementation Mapping process involves creating implementation protocols and materials to support the *promotoras*’ empowerment training within the Faith in Action study. A bilingual and bicultural research team collaborated with community members, including Faith in Action *promotoras* and FBO leaders, to develop training materials. Materials were reviewed by bilingual, bicultural team members and refined to ensure clarity, readability, and cultural appropriateness. In addition, *promotoras* with prior experience delivering similar interventions participated in pilot sessions and provided feedback on the content, format, and examples used, which informed further refinement of the materials. The team also received feedback on outlines and drafted materials from community advisory board members (e.g., FBO leaders) and co-investigators. The team members subsequently revised and finalized the materials. A bilingual *promotora* coordinator, who also delivered the standard training, was designated to lead the enhanced training sessions. They facilitated discussions, provided guidance, and ensured that the training equipped *promotoras* with the skills and knowledge needed to implement PA interventions in their communities. Involving community members, FBO leaders, and *promotoras* ensured that the materials grounded in the lived experiences and practical realities of those who would be implementing the interventions. For example, *promotoras* offered specific examples of situations where assertiveness or leadership skills were necessary, allowing the training to include role-playing exercises that reflected actual scenarios. By incorporating feedback from experienced *promotoras* and community members and appointing a dedicated coordinator, this approach ensured that the implementation protocols and materials were tailored to the specific needs and preferences of the target population, thereby potentially enhancing the success and sustainability of the Faith in Action study.

#### Task Five: Plan for Evaluation

An evaluation plan was developed to assess changes in *promotora* empowerment, skills, and implementation outcomes over time using interviewer-administered surveys, supervisor assessments, and qualitative feedback.

## Results

Results are presented by task consistent with Implementation Mapping.

### Task 1: Needs Assessment

Findings from the focus groups with *promotoras* informed the priorities for implementation strategies. More specifically, implementation strategies were informed by CFIR domains such as Inner Setting (the setting in which the innovation is implemented, e.g., hospital, school, city) and Individuals (the roles and characteristics of individuals; see Appendix A). While *promotoras* noted feeling equipped to lead PA classes and discuss health with community members, they lacked confidence and leadership skills to navigate the organizational context. More specifically, *promotoras* expressed concern with the lack of institutional FBO support (e.g., locked or unclean classrooms, difficulty scheduling) and many had a hard time maintaining positive rapport with FBO leaders or advocating for program needs. Other frequent problems *promotoras* encountered were difficulty motivating enrolled participants to attend, interpersonal disruptions that affected their classes (e.g., problems with participants), and conflict with other FBO programs or FBO needs and preferences (e.g., music too loud, non-ideal class times, dress code concerns). Results suggest that *promotoras* could benefit from planning and problem-solving skills to anticipate and overcome challenges, empowerment to take initiative in helping improve program fit with their FBO and community, and stronger interpersonal skills to advocate for program needs and maintain rapport with FBO leaders, staff, and participants.

### Task 2: Constructing Matrices

Results from task 2 identified two primary implementation outcomes for *promotoras* delivering Faith in Action within faith-based organizations: (1) successfully organizing and delivering physical activity sessions and (2) supporting participant engagement and retention (see Table 1). Performance objectives associated with program delivery included anticipating and addressing common implementation challenges, communicating program goals and logistical needs with faith-based organization leaders and staff, and advocating for program resources while establishing rapport with key organizational leaders. Performance objectives related to participant engagement and retention included motivating participants, responding to interpersonal challenges, maintaining regular supportive communication, and adapting program activities and messaging to fit the faith-based and community context while preserving core program components.

Matrices of change objectives were then constructed by crossing performance objectives with selected determinants to specify the precise changes required to support *promotora* implementation behavior (Table 2). Determinants included knowledge, skills, and self-efficacy relevant to each performance objective. For example, performance objectives related to advocacy required changes in knowledge of program goals and organizational context, as well as increases in communication skills and self-efficacy for engaging with faith-based organization leadership. Similarly, performance objectives related to anticipating and addressing implementation challenges required increased knowledge of common barriers and enhanced planning and problem-solving skills. Together, these matrices of change objectives identified priority targets for the *promotora* empowerment training and provided a structured foundation for selecting theory-informed methods and implementation strategies.

### Task Three: Select Theoretical Methods and Design Implementation Strategies

In task 3, theory-informed approaches were used to guide the selection and application of change methods to address the determinants identified in task 2. Specifically, Empowerment Theory (Zimmerman, 1990) and Dialectical Behavior Therapy (DBT; Linehan, 2025) interpersonal effectiveness skills served as organizing theoretical frameworks for identifying change methods relevant to *promotoras*’ implementation roles within faith-based organizations. These theories were selected due to their strong alignment with priority determinants in task 2, including self-efficacy, communication skills, and problem-solving capacity in organizational contexts.

Empowerment Theory conceptualizes empowerment as a multilevel process through which individuals develop self-efficacy, critical awareness, and the skills necessary to take effective action within social and organizational systems (Zimmerman, 1990). Drawing on Empowerment Theory, change methods emphasizing participation, active learning, individualization, and elaboration were prioritized to support *promotoras*’ development of agency, confidence, and leadership as community-based implementers. DBT is a skills-based cognitive-behavioral approach that emphasizes the development of practical skills in interpersonal effectiveness, emotion regulation, and adaptive problem-solving. DBT interpersonal effectiveness skills informed the selection of change methods related to guided practice, modeling, feedback, and skill rehearsal to strengthen assertive communication, perspective-taking, and *promotoras*’ ability to navigate interpersonal and organizational challenges within faith-based settings. These theory-informed change methods were subsequently operationalized into practical applications delivered through interactive, Spanish-language training sessions, including facilitated discussion, role-play, guided practice, and structured problem-solving exercises (Table 3).

### Task Four: Develop Implementation Protocols and Materials

In task 4, the final *promotora* empowerment training protocol and materials were produced based on the change methods and practical applications identified in task 3. The resulting training consisted of five structured, in-person training sessions, each lasting approximately 1 to 1.5 hours. Sessions were delivered in Spanish by the *promotora* coordinator and were designed to be highly interactive to support skill development and engagement. Each training session was supported by standardized PowerPoint slide decks that guided session content and facilitated consistent delivery across cohorts. Sessions incorporated a variety of interactive components, including guided discussions, skill-building activities, role-play exercises, and structured worksheets designed to reinforce key concepts and support application of skills to real-world implementation challenges. In addition to in-session materials, *promotoras* were provided with supplemental handouts and resources to retain after each session for ongoing reference and application during program implementation.

The final training protocol included five core sessions addressing priority implementation domains identified through Implementation Mapping: Communication & Conflict Resolution, Planning & Problem Solving, Motivating Others, Adapting the Program, and Social Media (Table 4). Each session was explicitly linked to specific implementation objectives, ensuring alignment between the training content and targeted implementation outcomes.

### Task 5: Formulating an Evaluation Plan

Task 5 of the Implementation Mapping process involves evaluating the outcomes of the *promotora* empowerment training within Faith in Action. We will assess changes in *promotora* empowerment and skills from baseline (pre-training) and several follow-ups (6 months, 12 months, and 18 months post-training) through interviewer-administered self-reported surveys and supervisor checklists. These self-report surveys assess competencies targeted in the first three training sessions (see Table 5 and Appendix B), including interpersonal and communication skills (Puggina & Silva, 2014; Wiemann, 1977; Session 1), problem-solving skills (Barkman & Machtmes, 2002; Session 2), confidence to promote physical activity (Haas et al., 2015; Mincemoyer et al., 2005; Session 3), and empowerment efficacy (Frans, 1993; Kasmel & Tanggaard, 2011). The three follow-up measurement points will also capture program adaptations made by *promotoras* (session 4) and *promotoras*’ use of social media in relation to program implementation (session 5). The three follow-up measurement points will also capture program adaptations *promotoras* make (session 4) and *promotoras*’ current use of social media in relation to program implementation (session 5). Additionally, the *promotoras*’ supervisor will assess their problem-solving and interpersonal skills throughout the program. During weekly check-ins, the coordinator will use a guided protocol to ask qualitative questions that target skills gained in all five training sessions (e.g., “How did you resolve them?”), recording responses in a database. Twice during the program, the coordinator will also rate each *promotora* on seven key skills (e.g., proactivity, planning ahead) using a 5-point Likert scale. Together, these skills are expected to mediate successful implementation, which is captured in the survey by asking about challenges with program implementation or FBO staff interactions. Finally, we will assess implementation outcomes (e.g., program fidelity and participant engagement) to understand how effectively *promotoras* are implementing Faith in Action. In post-training evaluations, *promotoras* will also share qualitative and quantitative feedback on the feasibility and acceptability of the training (e.g., satisfaction with the training content and delivery, perceived efficacy, and impact of the training in preparing them for their role).

## Discussion

This study addressed a notable gap in the field by utilizing Implementation Mapping to develop a *promotora* empowerment training with potential to be adapted for future interventions in similar FBOs or community organizations. Through a systematic approach guided by Implementation Mapping, a comprehensive training curriculum was created to empower *promotoras* to effectively address challenges they may face as program implementers. The study developed five key training sessions focusing on Communication & Conflict Resolution, Planning & Problem Solving, Motivating Others, Adapting the Program, and Social Media.

This training fills a critical research gap by addressing the organizational and implementation challenges that are often overlooked in traditional *promotora* training (Covert et al., 2019; Koskan et al., 2013a; Plescia et al., 2008; Rhodes et al., 2007). While most existing programs focus on enhancing *promotoras*’ abilities to connect with and support community members—emphasizing skills such as cultural competence, health education, and navigation—their capacity to navigate internal organizational barriers is frequently neglected (Covert et al., 2019; Koskan et al., 2013a; Plescia et al., 2008; Rhodes et al., 2007). As discussed, *promotoras* often face significant challenges within their organizations, including competing priorities, limited resources, poor communication, insufficient understanding of organizational procedures, and high staff turnover (Early et al., 2016; Otiniano et al., 2012; Sherman et al., 2017). This *promotora* empowerment training aims to address the implementation challenges faced within the specific context of FBOs, including the FBO’s culture of wellness, challenges in participant recruitment, the level of pastor involvement, availability of space for classes, and the ability to secure placement on the FBO calendar (Tettey, 2018). These challenges, which have been identified in previous studies (Early et al., 2016; Otiniano et al., 2012; Sherman et al., 2017), can severely impact the successful implementation and sustainment of health interventions (Koskan et al., 2013b, c). By incorporating modules specifically designed to address these organizational challenges—such as Communication & Conflict Resolution, Planning & Problem Solving, and Adapting the Program—this training provides *promotoras* with essential skills for overcoming these hurdles. This focus on professional and leadership skill development may better equip *promotoras* to manage and sustain their interventions within complex organizational environments, such as FBOs. Additionally, these modules provide a foundational framework that can be adapted for training community health educators to implement various health interventions beyond physical activity programs.

In addition to addressing gaps in *promotora* training content, this study clarifies the proposed mechanisms through which the *promotora* empowerment training is expected to influence implementation outcomes. Guided by Implementation Mapping, the training was intentionally designed a priori to target modifiable determinants of *promotora* implementation behavior, including skills, self-efficacy, leadership confidence, and communication capacity. Interactive training components (e.g., role-play, guided practice, and feedback) served as practical applications of theory-based change methods (e.g., modeling, guided practice, participation, elaboration) to strengthen these determinants. By engaging *promotoras* in realistic scenarios reflecting common organizational and interpersonal challenges, the training aimed to build confidence and competence in sub-behaviors critical for implementation, such as advocating for program resources, problem-solving scheduling or space constraints, motivating participants, and adapting program delivery while maintaining core components. Improvements in these *promotora* implementation behaviors are expected to support more consistent and effective delivery of Faith in Action physical activity sessions within faith-based organizations, thereby enhancing participant engagement and the potential for program impact.

Beyond the content of the training itself, the process used to develop the *promotora* empowerment curriculum addresses an additional gap in the literature related to stake-holder engagement in implementation strategy development (Rhodes et al., 2007). Although community-based health promotion emphasizes participatory approaches (Merzel & D’Afflitti, 2003), many *promotora* and community health worker training programs provide limited detail on how key partners are involved in the systematic development of implementation strategies (Koskan et al., 2013a; Rhodes et al., 2007). In this study, the intentional inclusion of *promotoras*, FBO leaders, and community advisory board members throughout the Implementation Mapping process helped ground the training in lived experience and organizational realities. This collaborative approach aligns with community-based health promotion principles and enhances the relevance and feasibility of implementation efforts in faith-based and community settings.

This study has limitations. First, the generalizability of findings is limited, as the training program was developed and implemented within a specific geographic, cultural, and program context. Second, the *promotora* focus groups on task 1 were conducted post-intervention and did not include all *promotoras*, potentially limiting the comprehensiveness of understanding of *promotora* needs. Third, the absence of reported outcomes, which will only be available upon study completion, limits the ability to definitively assess the program’s impact. Despite these limitations, the study has several notable strengths. First, it employed a systematic, theory-based approach to curriculum development using Implementation Mapping, ensuring that each aspect of the training was grounded in evidence-based strategies. Second, the study prioritized community engagement by involving a wide range of stakeholders, including *promotoras*, community advisory board members, and FBO leaders, in the design and refinement of the training. This collaborative process not only tailored the training to the specific cultural and organizational needs of the target population but it may also potentially increase ownership and the likelihood of sustainability. Finally, the focus on *promotora* empowerment, through skill-building in areas such as advocacy and communication, positions the program as a model for fostering long-term capacity within the community, potentially leading to broader health impacts beyond this intervention. These strengths underscore the study’s contribution to addressing health disparities in a culturally sensitive, community-driven approach, while also demonstrating the feasibility of adapting and implementing similar interventions in other settings.

## Conclusion

This study describes the development of a *promotora* empowerment training program designed to enhance the implementation and sustainment of PA interventions in FBOs. *Promotora*-led interventions, implemented across diverse organizational settings, have been widely used to promote health across multiple domains, including chronic disease prevention and management, mental health, nutrition, and cancer screening among Latina/o populations (Early et al., 2016; Gustafson et al., 2025; Luque et al., 2019; Perez et al., 2013). However, *promotoras* often face implementation challenges such as unfamiliarity with organizational procedures which hinder successful implementation. Despite these challenges, most *promotora* trainings focus on training *promotoras* in their connection and role serving community members, such as patient navigation and health education (Rhodes et al., 2007). To address these gaps, it is essential to create and evaluate training programs that equip *promotoras* with comprehensive professional skill development necessary for navigating complex organizational environments, such as interpersonal communication skills needed to advocate for program needs.

The *promotora* empowerment curriculum aims to not only improve the effectiveness of *promotora*-led initiatives but also to serve as a resource for developing future training programs that empower *promotoras* to overcome implementation barriers effectively. Although developed for a faithbased physical activity intervention for Latinas, the *promotora* empowerment training focuses on implementation competencies (e.g., problem-solving, communication, and organizational navigation) that are applicable across health behaviors, populations, and community settings, including other culturally tailored promotion and prevention efforts delivered through diverse faith-based and community organizations. By strengthening *promotoras*’ professional skills in organizational and interpersonal domains, this curriculum aims to enhance implementation capacity, which may support the effective and sustainable delivery of physical activity programs tailored for Latina communities. Through this initiative, we aim to address disparities in PA and promote holistic well-being among Latina populations, leveraging community strengths and cultural contexts to foster sustainable health improvements.

## Supplementary Material

11121_2026_1949_MOESM2_ESM

11121_2026_1949_MOESM1_ESM

The online version contains supplementary material available at https://doi.org/10.1007/s11121-026-01949-5.

## Figures and Tables

**Fig. 1:**
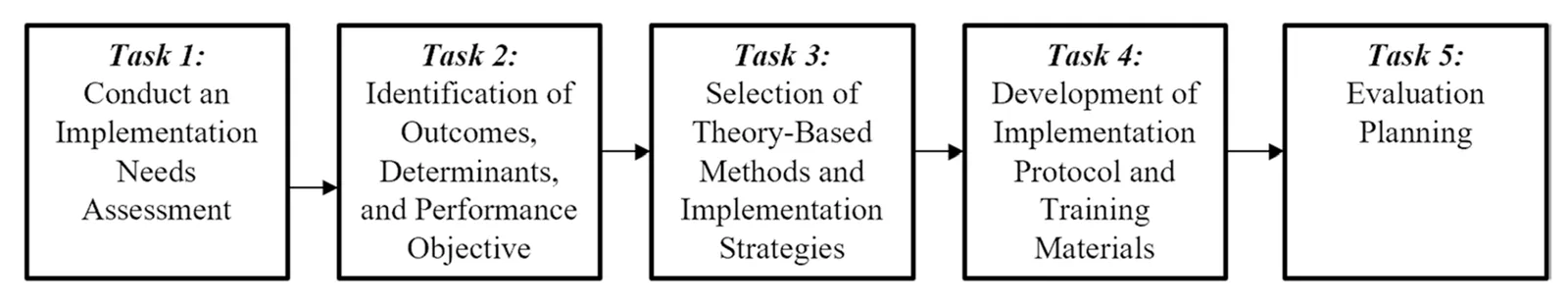
Implementation Mapping tasks

**Table 1:** Performance objectives and implementation outcomes for *promotoras* in program delivery

Implementation outcomes	Performance objectives
*Promotoras* successfully organize and deliver Faith in Action physical activity sessions within faith-based organizations	*Promotoras* anticipate common challenges and use problem-solving strategies during implementation *Promotoras* effectively communicate program goals and needs to FBO leaders and staff *Promotoras* are skilled in advocating for program needs and have established rapport with key organizational leaders
*Promotoras* support participant engagement and retention in Faith in Action PA sessions	*Promotoras* motivate participants and respond effectively to interpersonal challenges *Promotoras* maintain regular, supportive communication with participants and promote community engagement *Promotoras* adapt program activities and messaging to fit the FBO and community context without compromising core components

**Table 2:** Partial matrices of change objectives: key examples

Performance objectives	Knowledge	Skills & self-efficacy
Develop interpersonal skills to advocate for program needs and build rapport with key organizational leaders	Understanding of program goals, roles, and effectiveness	Demonstrating confidence in articulating program goals and effectiveness
	Knowledge of effective communication strategies	Demonstrating effective communication with FBO leaders and staff to build rapport
	Understanding of the importance of aligning program goals with FBO values, resources, or context	Demonstrating confidence in aligning program goals with FBO values
Develop planning skills to anticipate common problems and have problem-solving skills to overcome challenges	Knowledge of potential challenges to program implementation	Demonstrating ability to anticipate and plan for potential implementation challenges
	Understanding of effective problem-solving techniques	Demonstrating effective problem-solving skills in addressing implementation challenges

**Table 3:** Implementation intervention plan: key examples

Determinants/change objectives	Theoretical change methods	Practical applications
Understanding the importance of aligning program goals with FBO values, resources, or context	Discussion; participation; individualization; active learning, elaboration	Training session that includes facilitated group discussion, active participation through shared reflection and questions. Tailoring examples to each FBO’s context, interactive learning activities, and guided reflection exercises linking Faith in Action goals to FBO values and resources
Knowledge of effective communication strategies	Empathy training; individualization; active learning; tailoring	Training session that includes modeled demonstrations of effective, culturally responsive communication, facilitated discussion emphasizing perspective-taking and empathy, opportunities for *promotoras* to ask questions and practice communication strategies, and adaptation of examples to *promotoras*’ specific roles and FBO contexts
Demonstrate ability to anticipate and plan for potential implementation challenges	Scenario-based risk information; problem-posing education; participation; guided practice, feedback	Training using case-based scenarios depicting common implementation barriers, group discussion to identify barriers and potential solutions, collaborative participation in planning responses, rehearsal of planning strategies during structured exercises, and facilitator feedback from the *promotora* coordinator to refine proposed solutions
Demonstrating effective problem-solving skills in addressing implementation challenges	Elaboration; problem-posing education; participation; guided practice, feedback, implementation intentions	Training with structured exercises in which *promotoras* generate, rehearse, and refine solutions to real-world implementation challenges. Training should also include rehearsed responses through role-play and practice activities, received targeted feedback from the facilitator, reflections on why selected solutions would be effective, and development of concrete action plans specifying when, where, and how strategies would be applied in future situations

**Table 4:** *Promotora* training session objectives

Session	Session title	Objective
1	Communication & Conflict Resolution	Develop interpersonal skills to advocate for program needs and build rapport with key organizational leaders
2	Planning & Problem Solving	Develop planning skills to anticipate challenges and have problem solving skills to overcome challenges
3	Motivating Others	Develop skills to motivate participants and improve capacity to respond to interpersonal challenges
4	Adapting the Program	Tailor the program to enhance fit with their FBO and community
5	Social Media	Create/manage social media and build a strong online community to enhance social support and program communication with participants

**Table 5:** Alignment of *promotora* empowerment training session with evaluation measures

Training session	Corresponding components of evaluation
Communication & Conflict Resolution	Self-report surveys assessing communication skills (Wiemann, 1977) Weekly coordinator-documented check-ins assessing challenges with FBO staff and strategies used for resolution
Planning & Problem Solving	Self-report surveys assessing problem-solving skills (Haas et al., 2015; Mincemoyer et al., 2005) Weekly coordinator-documented check-ins assessing implementation problems encountered and resolved
Motivating Others	Self-report surveys assessing confidence to promote physical activity
Adapting the Program	Weekly coordinator-documented check-ins assessing program adaptations made during implementation
Social Media	Self-report surveys assessing current use of social media for program delivery and participant engagement (developed in-house)

## Data Availability

The data and research materials supporting the findings of this study are not publicly available but may be available from the corresponding author upon reasonable request.
