# Supplementary material for: Use of Implementation Mapping to Develop a Promotora Empowerment Training for a Faith-Based Physical Activity Program for Latinas
Source: Prev Sci. Author manuscript; Available in PMC 2026 Oct 26. (PMC13696355; doi:10.1007/s11121-026-01949-5)
Supplement: Supplementary file 1 [file NIHMS2205131-supplement-11121_2026_1949_MOESM1_ESM.docx]

**Supplementary Appendix B**

**Promotora Empowerment and Implementation Measures**

**Empowerment Efficacy**

*Adapted from Kasmel & Tanggard (2011) and Frans (1993)*

|  | Strongly Disagree | Disagree | Neutral | Agree | Strongly  Agree |
| --- | --- | --- | --- | --- | --- |
| I have the knowledge and skills to influence the church community |  |  |  |  |  |
| I have the ability to impact my church community in important ways |  |  |  |  |  |
| I have confidence in my capabilities to make needed changes in my church |  |  |  |  |  |
| I am able to affect the area in which I live |  |  |  |  |  |
| I can influence church community members to take actions on important issues |  |  |  |  |  |
| I have the knowledge and skills to gather information relevant to my church community |  |  |  |  |  |
| I know I can make a difference in my church community |  |  |  |  |  |
| I am certain I can overcome barriers to make changes in the church |  |  |  |  |  |
| I participate in church activities |  |  |  |  |  |
| I am involved in my church community |  |  |  |  |  |
| I volunteer for community projects |  |  |  |  |  |
| I think it is important for me to get involved in my church community |  |  |  |  |  |
| I feel that efforts to address community issues are worthwhile |  |  |  |  |  |
| I am motivated to get involved in my church community |  |  |  |  |  |
| I am usually able to think through all the relevant issues |  |  |  |  |  |
| I always know who has the power in different situations |  |  |  |  |  |
| My place in the world is always very clear to me |  |  |  |  |  |
| I usually know exactly where I stand |  |  |  |  |  |
| If I don’t have the answer to a question, I always know where to get it |  |  |  |  |  |
| It is helpful to join with others to solve problems |  |  |  |  |  |
| I can motivate others to help solve problems |  |  |  |  |  |

**Confidence to Promote Physical Activity**

*Adapted from Hass et al. (2015) and Mincemoyer et al. (2005)*

| I am confident that I can promote physical activity… | Not  confident | Slightly confident | Moderately confident | Very confident | Extremely confident |
| --- | --- | --- | --- | --- | --- |
| During or after church service |  |  |  |  |  |
| During church-related meetings |  |  |  |  |  |
| Through church dissemination platforms (e.g., social media, emails, bulletins, Flocknote) |  |  |  |  |  |

| I am confident that I can provide information about… | Not  confident | Slightly confident | Moderately confident | Very confident | Extremely confiden |
| --- | --- | --- | --- | --- | --- |
| The benefits of physical activity to church members |  |  |  |  |  |
| Overcoming barriers to physical activity |  |  |  |  |  |
| Goal-setting for physical activity |  |  |  |  |  |
| Monitoring physical activity |  |  |  |  |  |
| Resources in the community to be physically active |  |  |  |  |  |

**Solving Problems**

*Shortened from Barkman & Krisanna (2002)*

| How often do the following occur… | Never | Rarely | Sometimes | Often | Always |
| --- | --- | --- | --- | --- | --- |
| When I have a problem, I first figure out exactly  the problem is |  |  |  |  |  |
| I try to get all the facts before trying to solve a problem |  |  |  |  |  |
| When I have a problem, I look at what is and what should be. |  |  |  |  |  |
| I look ahead and try to prevent problems before they happen |  |  |  |  |  |
| When faced with a problem, I try to determine what caused it. |  |  |  |  |  |

| How often do the following occur… | Never | Rarely | Sometimes | Often | Always |
| --- | --- | --- | --- | --- | --- |
| When solving a problem, I look at all possible solutions |  |  |  |  |  |
| I look at the likely results for each possible solution |  |  |  |  |  |
| After choosing a solution, I put it into action. |  |  |  |  |  |
| When a solution is not working, I try to figure out what is wrong |  |  |  |  |  |
| Once I have solved a problem, I step back to see how my solution is working. |  |  |  |  |  |
| If my solution is not working, I will try another solution |  |  |  |  |  |

**Communicative Competence Scale (CCS)**

*Shortened from* [*Wiemann*](https://www.drugsandalcohol.ie/26794/1/Communicative%20Competence%20scale.pdf) *(1997)*

| Indicate your agreement with each item by indicating that response for each statement. | Strongly Disagree | Disagree | Neutral | Agree | Strongly  Agree |
| --- | --- | --- | --- | --- | --- |
| I treat people as individuals. |  |  |  |  |  |
| I can deal with others effectively. |  |  |  |  |  |
| I am a good listener. |  |  |  |  |  |
| I won’t argue with someone just to prove I am right. |  |  |  |  |  |
| I ignore other people’s feelings. |  |  |  |  |  |
| I listen to what people say to me. |  |  |  |  |  |
| I generally know what type of behavior is appropriate in any given situation. |  |  |  |  |  |
| I usually do not make unusual demands on my friends. |  |  |  |  |  |
| I can easily put myself in another person’s shoes. |  |  |  |  |  |
| I am interested in what others have to say. |  |  |  |  |  |
| I am not afraid to speak with people in authority. |  |  |  |  |  |
| I generally say the right thing at the right time. |  |  |  |  |  |
| I am sensitive to others’ needs of the moment. |  |  |  |  |  |

**Your Use of Social Media**

*Project-developed*

| Which social media platforms did you create or offer to your church for communication? (Please select all that apply): | □ Facebook   □ Instagram  □ WhatsApp  □ FlockNote  □ None  □ Other (please specify): |
| --- | --- |

| How frequently do you engage with others on the social media you’ve created using the following features? | Infrequently | Monthly | Weekly | Daily |
| --- | --- | --- | --- | --- |
| Send messages |  |  |  |  |
| Have voice or video calls |  |  |  |  |
| Post status updates |  |  |  |  |
| Multimedia sharing (e.g. post photos/videos) |  |  |  |  |

| For what purposes do you use the social media platform created for your church? (Select all that apply): | □ Sending updates on events or activities  □ Connecting participants with each other  □ Sending educational content  □ Seek support from others  □ Sharing your or others’ experiences  □ None  □ Other (please specify): |
| --- | --- |
