# Supplementary material for: Use of Implementation Mapping to Develop a Promotora Empowerment Training for a Faith-Based Physical Activity Program for Latinas
Source: Prev Sci. Author manuscript; Available in PMC 2026 Oct 26. (PMC13696355; doi:10.1007/s11121-026-01949-5)
Supplement: Supplementary file 2 [file NIHMS2205131-supplement-11121_2026_1949_MOESM2_ESM.docx]

**Supplementary Appendix A**

**Task 1 Focus Group Findings**

**Table A1**

*Key Focus Group Findings Organized by CFIR Constructs Informing Implementation Strategy Development*

| **CFIR Construct (CFIR domain)** | **Theme** | **Exemplar Quote** |
| --- | --- | --- |
| **Available Resources (Inner setting)** The level of resources dedicated for implementation and ongoing operations, including money, training, education, physical space, and time. | **Support:** Resources and assistance given to ensure the success of the program. | “JM: we go to a room full of chairs... we have pictures, you also have access to those pictures where, at St. John of the Cross they used the room, they were participating and we were in a little corner teaching yoga classes in the middle of tables.” |
| **Implementation Climate (Inner setting)** The absorptive capacity for change, shared receptivity of involved individuals to an intervention, and the extent to which use of that intervention will be rewarded, supported, and expected within their organization. | **Support:** Resources and assistance given to ensure the success of the program. | “KF: In the case that I came in first that my colleague LG, (inaudible) and I remember I interacted with the deacon. The deacon agreed but not, honestly, he sort of took it lightly and I remember that he told me that the program was not going to prosper. He said: I don’t think the community here is going to get interested because at some point we tried to bring some English classes and some other types of classes and the community didn’t respond.” |
| **Compatibility (Inner setting)** The degree of tangible fit between meaning and values attached to the intervention by involved individuals, how those align with individuals’ own norms, values, and perceived risks and needs, and how the intervention fits with existing workflows and systems. | **Participant Engagement:** Participant’s investment or dedicated participation in the program. | “AF: Later on, they did, they did provide it, but one or two out of forty came regardless. So, it was like…there were not that many people.” |
| **Networks & Communications (Inner setting)** The nature and quality of webs of social networks and the nature and quality of formal and informal communications within an organization. | **Church Culture:** The underlying beliefs, assumptions, values, and ways of interacting that contribute to the unique social and psychological environment of the church. | “KF: I remember now, as AH also says, sometimes there’s conflict between the administrative staff that works in the church and the deacon himself or the priest. So, in my case at MC it was very hard; in fact, I even remember that we went to the deacon and we had a meeting with the deacon and a whole schedule was set with times and everything, and then I got a phone call from the secretary, she was very upset, saying why I was setting up schedules without her authorization, but the deacon was the one that gave me authorization first, right?” |
| **Self-Efficacy (Characteristics of individuals)** Individual belief in their own capabilities to execute courses of action to achieve implementation goals. | **Promotora Characteristics:** Traits, abilities, and assets of the *promotora* that impacted her ability to do her job. | “AH: For me the participants’ enthusiasm to exercise was easier for me, right? And when I was teaching a class and they wanted more and more, and they always wanted a change of music, they always wanted strenuous exercise. So, that kept me with them, uh….at 100% right?” |
